# Editorial: The emerging role of metabolism and metabolic-related receptors on neutrophil extracellular traps (NET) formation

**DOI:** 10.3389/fimmu.2022.1028228

**Published:** 2022-09-26

**Authors:** Rafael Agustín Burgos, Dirk Werling, Carlos Rodrigo Hermosilla

**Affiliations:** ^1^ Laboratory of Inflammation Pharmacology and Immunometabolism, Faculty of Veterinary Sciences, Institute of Pharmacology and Morphophysiology, Universidad Austral de Chile, Valdivia, Chile; ^2^ Centre for Vaccinology and Regenerative Medicine, Department of Pathobiology and Population Sciences, Royal Veterinary College, Hatfield, United Kingdom; ^3^ Institute of Parasitology, Biomedical Research Center Seltersberg (BFS), Justus Liebig University Giessen, Giessen, Germany

**Keywords:** NET (neutrophil extracellular traps), inflammation, innate immunity, immunometabolism, cellular reprogramming

Neutrophils play a central role in host innate immunity as first cell line to fight pathogens; however, they are also being responsible for “collateral damage”. Neutrophils have a wide repertoire of biological responses, including reactive oxygen species (ROS)-dependent and -independent effector mechanisms. Release of neutrophil extracellular traps (NET) has been added as key innate immune reaction. Understanding NET formation has broadened our knowledge regarding innate defence mechanisms as well as pathological mechanisms associated with occurrence of uncontrolled NET.

Biological processes associated with neutrophil responses are closely linked to cellular metabolism. Glycolysis seems to be the main route associated with ATP generation, phagocytosis, and NET (1, 2), and the low abundance of mitochondria in neutrophils has led to the assumption that neutrophils exclusively rely on glycolysis for their biological functions (3). However, different metabolic routes are required to fulfil energetic, biosynthetic, and functional requirements of activated neutrophils, including TCA cycle, oxidative phosphorylation (OxPhos), pentose phosphate pathway (PPP), and fatty acid oxidation (FAO) (4). These metabolic pathways can generate metabolites that exert second messenger functions or act as ligands of receptors (e.g. purinergic receptors, succinate receptor SUCNR1 and lactate receptor HCA2) (5, 6).

Present ‘Research Topic’ mainly discusses advanced investigations on metabolic signatures associated with NET and with their key roles in various pathological disorders, infective diseases and morbidities. Distinct neutrophil differentiation status, activation as well as metabolic adaptations are needed, all of them being finely regulated, before neutrophils can activate their effector mechanisms. Consistently, metabolic reprogramming of neutrophils is induced by inflammatory mediators, pathogens and soluble factors released in several pathologies. In fact, metabolic changes can drastically affect neutrophil-derived responses to pro-inflammatory agents or to antigens from viruses, bacteria, fungi, protozoa and large parasites leading to NET. Conejeros et al., 2022, support new evidence on key role of metabolic events in apicomplexan *Eimeria bovis*-triggered NETosis. Sporozoites of *E. bovis* triggered release of ‘anchored’ NET (i. e. connected to disrupted PMN) - and ‘cell-free’ NET (i. e. without contact to disrupted PMN) phenotypes and both being dependent on ATP synthase and LDH activity. Authors propose purinergic receptor P2X1 as relevant signaling pathway in *E. bovis*-induced vital NETosis (7). This support previous evidence on critical role of mitochondrial metabolism, ATP release and purinergic receptors in NET (8, 9). In addition, increased LDH activity could promotes metabolism of pyruvate to lactate, the latter being a well-known inflammatory modulator (6).

Stojkov et al., 2022, review key metabolic pathway of neutrophils associated with increase in NET. Several diseases, including obesity, diabetes, cancer, SLE, rheumatoid arthritis (RA), sepsis, cystic fibrosis (CF), and COVID-19 result in metabolic shifts of neutrophils toward glycolysis resulting in increased NET (10). The alternative glucose-dependent metabolic pathway is the pentose-phosphate pathway (PPP), also known as hexose monophosphate shunt, being involved in NADPH oxidase (NOX)-dependent ROS production, and consequently contributing to NET. Several metabolic changes which favour glycolysis have been described in neutrophils. Enhanced hypoxia inducible factor-1α (HIF-1α) in neutrophils lead to expression of LDH, PDKs (pyruvate dehydrogenase kinases), and GLUT1, thereby increasing pro-inflammatory functions and NET release during RA and COVID-19 (Stojkov et al.).

Indeed, metabolism disturbances and NET are closely related in airway diseases. Moran et al., 2022 here show that release of NET is a pathognomonic feature of pulmonary inflammation in patients suffering from CF. In this disease, a metabolic reprogramming of neutrophils associated with an increase in mTOR-dependent pathway favouring greater re-expression of GLUT1 thereby resulting in glucose consumption (11). In addition, COVID-19 infection induces accumulation of neutrophils in affected lung evidencing pivotal role of uncontrolled NET associated with a greater clinical severity (12). Herein, role of glucose metabolism has been proposed as key event in NET formation of COVID-19-infected patients with hyperglycaemia (e. g. diabetes) (13), suggesting that neutrophil-mediated metabolism plays an important role in pathogenesis of COVID-19 infections.

Evidence suggests that the hyperglycaemia observed in patients with type II diabetes could increase release of NET (14); however, this does not seem to be to the same extent in patients with type I diabetes mellitus (T1DM). Aukrust et al., 2021, conduct a cross-sectional study of patients with long-term T1DM and age-matched controls, showing that NET levels were not associated with presence of T1DM or glycaemic status, and neither did markers of NET in coronary artery disease in patients with T1DM. An alteration in neutrophil function associated with a reduction in NET formation in T1DM should be studied in future studies, which will help to clarify the role of metabolism in T1DM-induced NETosis (15).

During metabolic reprogramming in macrophages, a disruption of TCA cycle has been detected, which leads to accumulation of citrate, transforming it into itaconate. Itaconate is a potent anti-inflammatory agent, produced by macrophages and it opens up new possibilities for development of therapeutic tools to efficiently control inflammation (16). Indeed, derivatives developed from itaconate, such as 4-octyl itaconate (4-OI) and dimethyl itaconate (DI) have a greater membrane permeability and can be transformed into itaconate inside cells by esterases (17). Burczyk et al. 2022, describe that 4-OI reduces NET formation, with HIF-1α downregulating the expression of 4-OI-pre-treated LPS-stimulated neutrophils. The authors suggest that blockage of HIF-1α by a specific inhibitor diminishes NET formation, as does inhibition by 4-OI (18). Indeed, stabilization of HIF-1α in neutrophils through release of mitochondrial ROS (mROS) is a critical process in NET extrusion (Burczyk et al., 19), and mROS is a potent intracellular signal triggering NET release (20, 21). In addition, neutrophils stabilize HIF-1α through mROS *via* glycerol-3-phosphate pathway (19). Thus, the article of Burczyk et al. 2022 suggests that NET release can be reduced using new derivatives originating from cellular metabolic pathways.

Overall, collected articles in this ‘Research Topic’ comprehensively summarize critical role of metabolism during NETotic process. Key molecules and signalling pathways participating in neutrophil metabolism are also discussed. We hope to improve discussion on this neglected topic and hopefully inspiring new investigations on pivotal role of metabolism during NET release, and further to contribute to development of novel therapeutic approaches for the control of NET in several infective diseases and morbidities.

## Author contributions

RB contributed to the concern and design of this editorial. CH and DW contributed to the writing of the article. CH and DW revised the manuscript. All authors contributed to the article and approved the submitted version.

## Funding

The present research was supported by the Chilean “Fondo Nacional de Desarrollo Científico y Tecnológico” (FONDECYT; No 1210754 awarded to RB) and the German “Bundesministerium für Bildung und Forschung” (BMBF; DLR 01DG20023 awarded to CH).

## Conflict of interest

The authors declare that the research was conducted in the absence of any commercial or financial relationships that could be construed as a potential conflict of interest.

## Publisher’s note

All claims expressed in this article are solely those of the authors and do not necessarily represent those of their affiliated organizations, or those of the publisher, the editors and the reviewers. Any product that may be evaluated in this article, or claim that may be made by its manufacturer, is not guaranteed or endorsed by the publisher.
